# Gut microbial composition associated with risk of premature aging in women with Yin-deficiency constitution

**DOI:** 10.3389/fcimb.2024.1500959

**Published:** 2025-01-03

**Authors:** Yingying Zhai, Jing Li, Yanqi Cao, Yufei Li, Xuejie Bao, Jinfeng Liang, Qi Liu, Yifan Xia, Ruoxi Yu

**Affiliations:** ^1^ National Institute of Traditional Chinese Medicine Constitution and Preventive Medicine, Beijing University of Chinese Medicine, Beijing, China; ^2^ School of Traditional Chinese Medicine, Beijing University of Chinese Medicine, Beijing, China

**Keywords:** gut microbiota, premature aging, machine learning, traditional Chinese medicine, Yin-deficiency constitution

## Abstract

**Background:**

Yin-deficiency constitution (YinDC) refers to a traditional Chinese medicine concept, characterized by an imbalance state that includes an imbalance in the gut microbiota, resulting from a relative deficiency of Yin fluids within the body. In recent years, it has become apparent that the composition and structure of the gut microbiota play a significant role in the aging process. The imbalance of gut microbiota in YinDC may accelerate the aging process. However, the specific gut microbiota compositions involved in the YinDC premature aging process remain unknown.

**Methods:**

In this study, we conducted a cohort study including 60 women with YinDC and BC to analyze their gut microbiota composition. We integrated 16S rDNA sequencing with machine learning methods to reveal the association between gut microbiota and premature aging in YinDC women.

**Results:**

We found a significant difference in the composition of gut microbiota between the YinDC and the BC group. At the phylum level, Cyanobacteria and Synerobacteria only emerged in the YinDC group. At the genus level, *Bacteroides, Bifidobacterium, Haemophilus, Alistipes*, and *Dialister* showed higher abundance in the YinDC group. *Bilophila, Eubacterium*, and *Aeromonas* were the most significant indicators influencing the YinDC premature aging. The YinDC group had the most functional gene pathways associated with the metabolism.

**Conclusion:**

Our study demonstrated that the gut microbiota was associated with premature aging in women with YinDC, potentially providing preliminary evidence and guidance for personalized anti-aging strategies.

## 1 Introduction

Aging is an inevitable life process of human beings, strongly associated with an increased risk of developing various chronic diseases (Guo et al., 2022). Delaying the aging process and preventing aging-associated chronic diseases have become a priority of current biomedical research. However, aging has different manifestations (Ahadi et al., 2020). Due to the heterogeneity of the aging process at the individual level, anti-aging research and clinical implementation are facing great challenges. Due to the individual differences in the aging process, the traditional Chinese medicine (TCM) constitution provides a feasible solution for individualized anti-aging. TCM constitution classifies the population into nine basic constitution types, which have different characteristics (Li et al., 2021). The association between constitution types and the aging process varies (Yu, 2020). Therefore, it is practically significant to conduct individualized anti-aging research based on the association between constitution and aging.

Yin-deficiency constitution (YinDC) is the predominant constitution type associated with aging, and accounts for the largest proportion of all biased constitution types in the elderly over 65 years old (Bai et al., 2020). YinDC plays an important role in the aging, and is associated with the occurrence of many geriatric diseases (Yu et al., 2015). YinDC is closely associated with the onset of female premature aging. Perimenopause is a special stage of physiological aging in women, and YinDC is the most common TCM Constitution type in women with perimenopausal syndrome (Li and Zhou, 2023). Due to the yin fluid deficiency in the body, women with YinDC are more likely to show aging characteristics such as dry skin and deafness. These symptoms are consistent with perimenopausal symptoms (Wang, 2021).

Currently, the research on mechanisms of premature aging caused by YinDC is still ongoing, and gut microbiota is an important part of these mechanisms. However, the specific mechanism of aging in YinDC caused by gut microbiota is not well explained. Existing evidence suggests that changes in the composition and function of gut microbiota accelerate the aging process and contribute to the development of chronic diseases in the elderly (Han et al., 2019; Yao and Li, 2007). In the previous study, it has been confirmed that the gut microbiota is associated with the aging process in YinDC (Liu Q. et al., 2020). The imbalance of gut microbiota in YinDC is involved in the aging process from multiple mechanisms. For example, the inflammatory factors (Yu et al., 2021) and gut microbiota (Yu et al., 2018) in YinDC are involved in the regulatory mechanism of the Nuclear factor-kappaB (NF-kappaB) aging signaling pathway. However, the corresponding bacterial species involved in the co-variation regulatory mechanism are not entirely clear.

To our knowledge, the identification of specific gut microbiota of YinDC is still lacking. To identify characteristic indicators associated with the YinDC premature aging, we choose genome research to study the composition of gut microbiota and apply machine learning methods to screen complex features from high-throughput genomic data (Chen et al., 2021). The combination of genome research and machine learning provides more powerful tools and more evidence for studies of aging-associated gut microbiota.

In this study, we measured the mRNA expression levels of gut microbiota in women aged 35 to 49 years with YinDC or Balanced constitution (BC). Then, we used the genomic method to analyze the factors associated with premature aging in YinDC and machine learning methods to construct the predictive model and identify the effective indicators of the gut microbiota in aging. This study has the following contributions: we show the composition and function of specific gut microbiota of premature aging in YinDC. We also confirmed the important indicators for the identification of premature aging in YinDC.

The rest of this article is organized as follows. In the Methods Section, research data, study design, and concrete statistical analysis methods are described. In the Results Section, we compared the composition and functional features of the gut microbiota between the YinDC and BC groups, and screened for the important indicators associated with premature aging in YinDC women. At last, we briefly discussed this work and the research plans in the Discussion Section.

## 2 Methods

### 2.1 Participants

60 healthy women aged 35 to 49 were recruited as participants for this control experiment according to medical ethics regulations. Patients eligible for the study had to meet the following requirements: biologically female, aged 35-49 years old, undergoing routine physical examination with no specific disease diagnosis, not pregnant or preparing for pregnancy, and in line with the criteria of YinDC or BC listed in the judgment standards of the TCM constitution issued by the Chinese Medicine Association of China in 2009 (ZZYXH/T157-2009). Additionally, participants were required to have a single constitution type of either YinDC or BC, rather than a combined constitution type. Ethical approval of the study was obtained from the Medical Ethics Committee of Beijing University of Chinese Medicine, and all participants signed an informed consent form.

Inclusion criteria: (a) Comply with the criteria for YinDC or BC; (b) Female, ages 35-49 years old; (c) Conducting routine physical examinations in the hospital instead of visiting for any specific disease diagnosis or treatment, and not taking any medications or supplements; (d) Signed the project informed consent form. Exclusion criteria: (a) Neither YinDC nor BC; (b) Have apparent physical disease or take any medications or supplements; (c) Women who are pregnant or are preparing for pregnancy; (d) Did not sign the informed written consent.

### 2.2 Sample collection and 16S sequencing

Participants were informed to avoid alcohol and tiredness the day before sample collection. Fresh stool samples collected were frozen overnight in liquid nitrogen and preserved in a -80 °C refrigerator. Blood samples collected in non-anticoagulant tubes are allowed to stand for 60 minutes at room temperature before being centrifuged at 3000rpm for 10 minutes. The supernatant is then aspirated, aliquoted, and stored at -80°C. Subsequently, stool samples were delivered to Shenzhen Microbiota Technology Co. for high-throughput sequencing of the same batch. Blood samples were delivered to Beijing Jinhaikeyu Technology Co. for blood biochemical index tests.

Genomic DNA was extracted from samples using the CTAB/SDS method, and its concentration and purity were monitored on a 1% agarose gel. The DNA was then diluted to 1 ng/µL. For sequencing, fecal DNA was used as a template to amplify the V3-V4 region of 16S rRNA using universal bacterial primers 515F (5’-GTGCCAGCMGCCGCGGTAA-3’) and 806R (5’-GGACTACHVGGGTWTCTAAT-3’). PCR reactions were performed with Phusion^®^ High-Fidelity PCR Master Mix. Detection was oprerated on a 2% agarose gel through electrophoresis and purified using the GeneJETTM Gel Extraction Kit (Thermo Scientific). Sequencing libraries were generated using the Ion Plus Fragment Library Kit 48 rxns (Thermo Scientific) and assessed on a Qubit@ 2.0 Fluorometer (Thermo Scientific). The library was sequenced on the Illumina Miseq PE300 platform, and 400 bp/600 bp single-end reads were generated by Shenzhen Micro Ecological Technology Co., LTD.

Total RNA from blood samples was extracted using Trizol reagent, (Aidlab Biotechnologies Co., Ltd., Beijing, China) and reverse-transcribed with the HiScript Reverse Transcription Kit (Vazyme Biotech Co., Ltd., Nanjing, China) for qRT-PCR analysis. The relative expression levels of TAK1, NFKBIA, CCL4, BCL2A1, and IL-8 mRNA were assessed using SYBR Green Real-Time PCR (Applied Biosystems, MA, US). Data were analyzed using the 2-ΔΔCt method, with each sample tested in triplicate.

### 2.3 Microbiome analysis using QIIME

The Final Effective Tags were obtained from the original sequencing data after splicing, quality control, and removal of chimera sequences. QIIME was applied to perform microbiome analysis (Caporaso et al., 2010). After the paired reads were spliced into one sequence, Operational Taxonomic Units (OTUs) were generated by the appliance of USE-ARCH (v7.0.1090) to merge the sequence with a more than 97% similarity. Based on the OTU cluster analysis results, we applied Venn Graph, α diversity analysis, and Beta diversity analysis to evaluate the diversity of the sample gut microbiota. With the appliance of the RDP Classifier, the representative sequences were compared with the SILVA database. The OTUs were identified with a confidence threshold of 0.8 to obtain the specific composition of the two groups of samples at phylum and genus classification levels.

### 2.4 Machine learning methods for identifying the feature gut microbiota

After analyzing the differences in the abundance and the diversity of gut microbiota between YinDC and BC, we screened the feature gut microbiota further. To construct predictive models and identify feature gut microbiota, we employed four machine learning methods widely used in healthcare and bioinformatics: Logistic Regression (LR), Random Forest (RF), Support Vector Machine (SVM), and eXtreme Gradient Boosting (XGBoost), each with its unique strengths. The combined use of these methods helps us to comprehensively understand the impact of gut microbiota on YinDC aging, thereby enhancing the reliability of our research findings. Based on the predictive models that were built, we further applied the Least Absolute Shrinkage and Selection Operator (Lasso) Regression, RF, and XGBoost to identify the feature gut microbiota influencing YinDC aging. Using multiple methods instead of a single approach ensures a more comprehensive identification process and reduces the likelihood of omitting the feature gut microbiota.

### 2.5 Functional enrichment analysis

To study the functional characteristics of gut microbiota in the group of YinDC, we used Tax4Fun to extract the prokaryotic whole-genome 16S rRNA gene sequences from the KEGG database and aligned the sequences to the SILVA SSU Ref NR database (BLAST bitscore > 1500) using the BLASTN algorithm to establish a correlation matrix. Function annotation of the SILVA database was achieved by mapping the KEGG database’s prokaryotic whole-genome functional information, annotated by UProC and PAUDA, to the SILVA database. We clustered the sequenced samples into OTUs using the SILVA database sequence as a reference. The resulting OTUs were then annotated with functional information and visualized using a functional annotation Venn Graph, a functional clustering heatmap, and a metabolic pathway diagram for multiple samples.

### 2.6 Statistical analysis

We performed statistical analysis using the SPSS 19.0 statistical software (SPSS Inc., Chicago, IL, USA). Quantitative variables were expressed as mean ± standard deviation. We used an independent two-sample t-test for the difference analysis between groups. Differences were considered statistically significant at P < 0.05. We used R software (4.1.1) to draw relative abundance histograms and construct predictive models. We performed a comparative test of the sequence quantity (i.e., absolute abundance) of taxa at the phylum and genus levels between groups using Mothur software (1.30.0). The Metastats statistical algorithm was applied to measure the differences in gut microbiota structure and identify significantly different species. The machine learning models were built by R 4.1.1 for logistic regression, random forest (4.6-14), e1071 (1.7-2), and XGBoost (1.7.5.1). Area under the curve (AUC) of the receiver operating characteristic (ROC) curve was used to evaluate the predictive performance of the models, using the R package pROC (1.18.0).

## 3 Results

### 3.1 Clinical characteristics of participants

The baseline statistics of the two groups of participants were shown in **Table 1**. Participants in the YinDC group were significantly younger than those in the BC group (P < 0.05), and there was no statistically significant difference in weight between the two groups (P ≥ 0.05).

**Table 1:** Age and weight by the BC and YinDC groups.

	BC group (n = 30)	YinDC group (n = 30)	P-value
Age (years)	44.27 ± 3.59	39.27 ± 3.77	*<* 0.05
Weight (kg)	55.40 ± 5.70	53.50 ± 5.44	*≥* 0.05

### 3.2 Comparison of gut microbiota between the YinDC and BC groups

**Figure 1:**
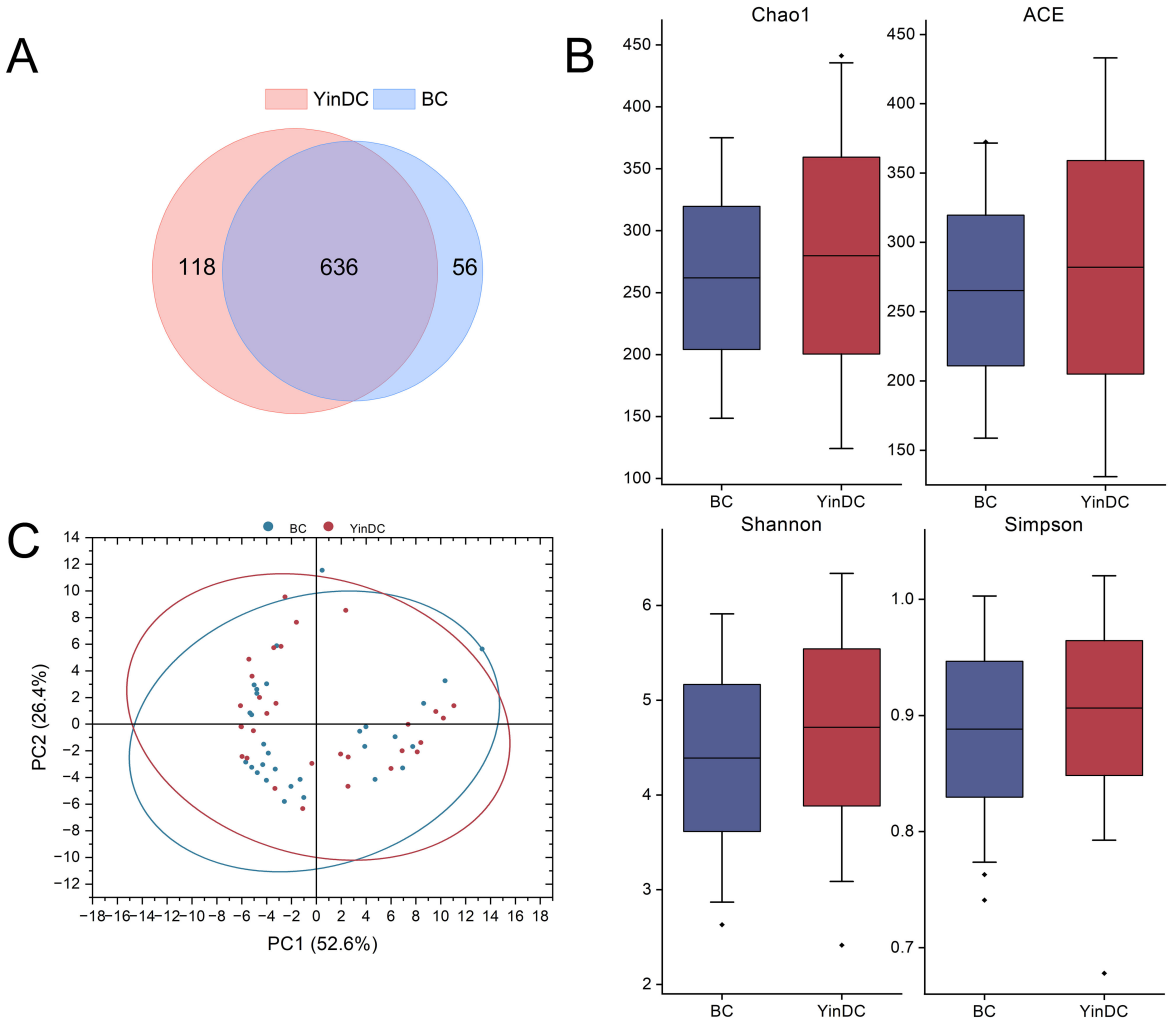
Diversity within and between groups. **(A)** Venn graph showing different OTU compositions of the two groups. **(B)** Box plot showing the differences of Chaol, ACE, Shannon, and Simpson indexes between the two groups. **(C)** The PCOA on weighted Unifrac distances shows global differences between the BC and YinDC groups.

We analyzed stool samples using 16S rDNA sequencing and clustered them into OTUs with 97% identity. A Venn diagram revealed that the BC and YinDC groups shared 636 OTUs, with 56 unique to the BC group and 118 unique to the YinDC group (**Figure 1A**).

Alpha diversity, assessed using Chao1, ACE, Shannon, and Simpson indexes, showed that while the YinDC group had higher richness and diversity, differences between the groups were not statistically significant (**Figure 1B**). Beta diversity, measured by the weighted Unifrac distance, demonstrated distinct differences in gut microbiota composition between the groups, as illustrated by principal coordinates analysis (PCoA) (**Figure 1C**).

**Figure 2:**
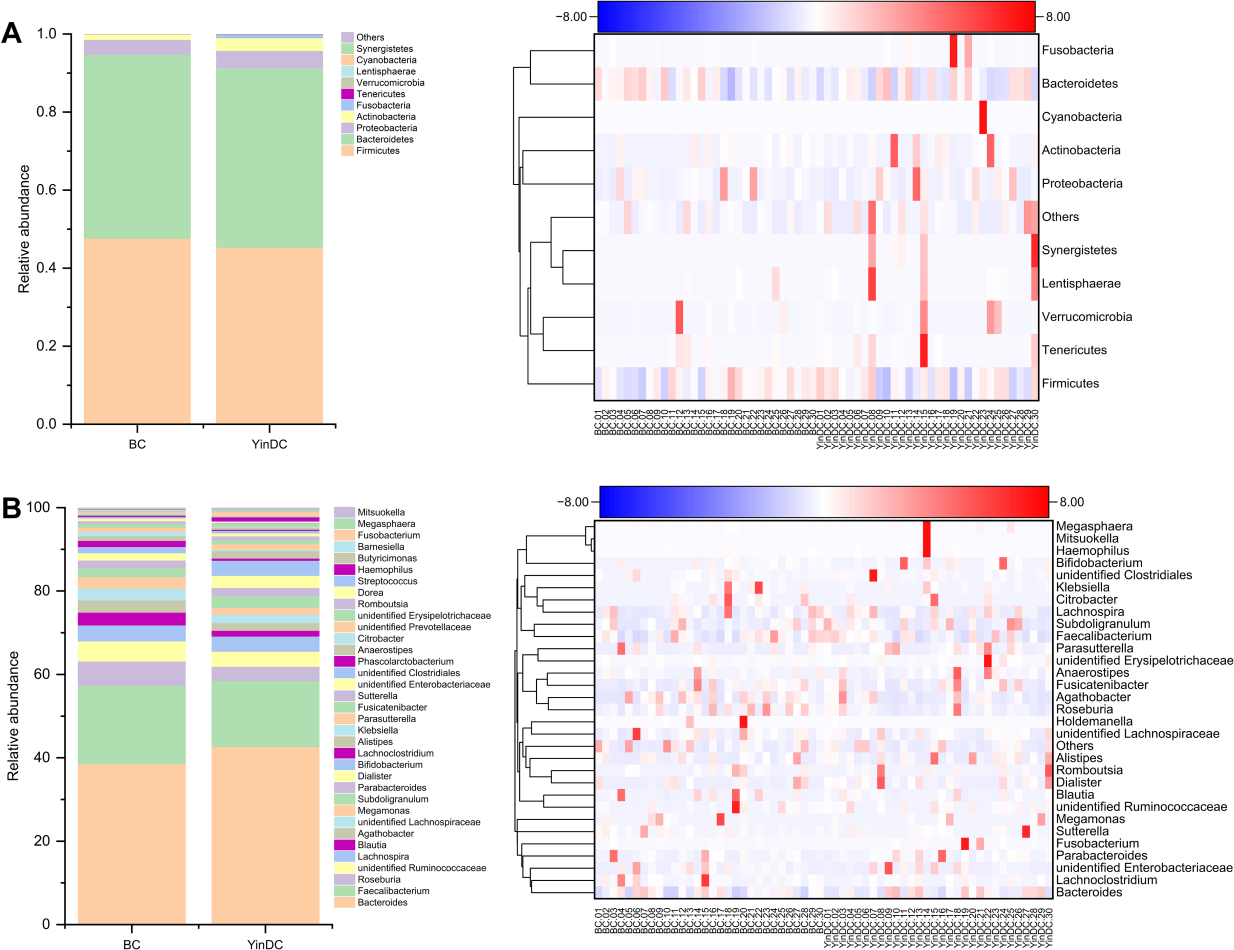
Column chart and heatmap illustrating the differential composition between the BC and YinDC groups at the phylum and genus levels. The top 10 at the phylum level and the top 30 at the genus level were selected based on average abundance.

To illustrate the differential composition of gut microbiota between the BC and YinDC groups, we selected the top 10 at the phylum level and the top 30 at the genus level to generate a column chart and a heatmap based on their average abundance (**Figure 2**). At the phylum level, Bacteroidetes and Firmicutes were predominant in both groups but were less abundant in the YinDC group. Other phyla exhibited higher abundance in the YinDC group with Cyanobacteria and Synergistetes being unique to it. At the genus level, the YinDC group had increased abundances of *Bacteroides*, *Bifidobacterium*, *Haemophilus*, *Alistipes*, and *Dialister*, whereas other genera were less prevalent.

**Figure 3:**
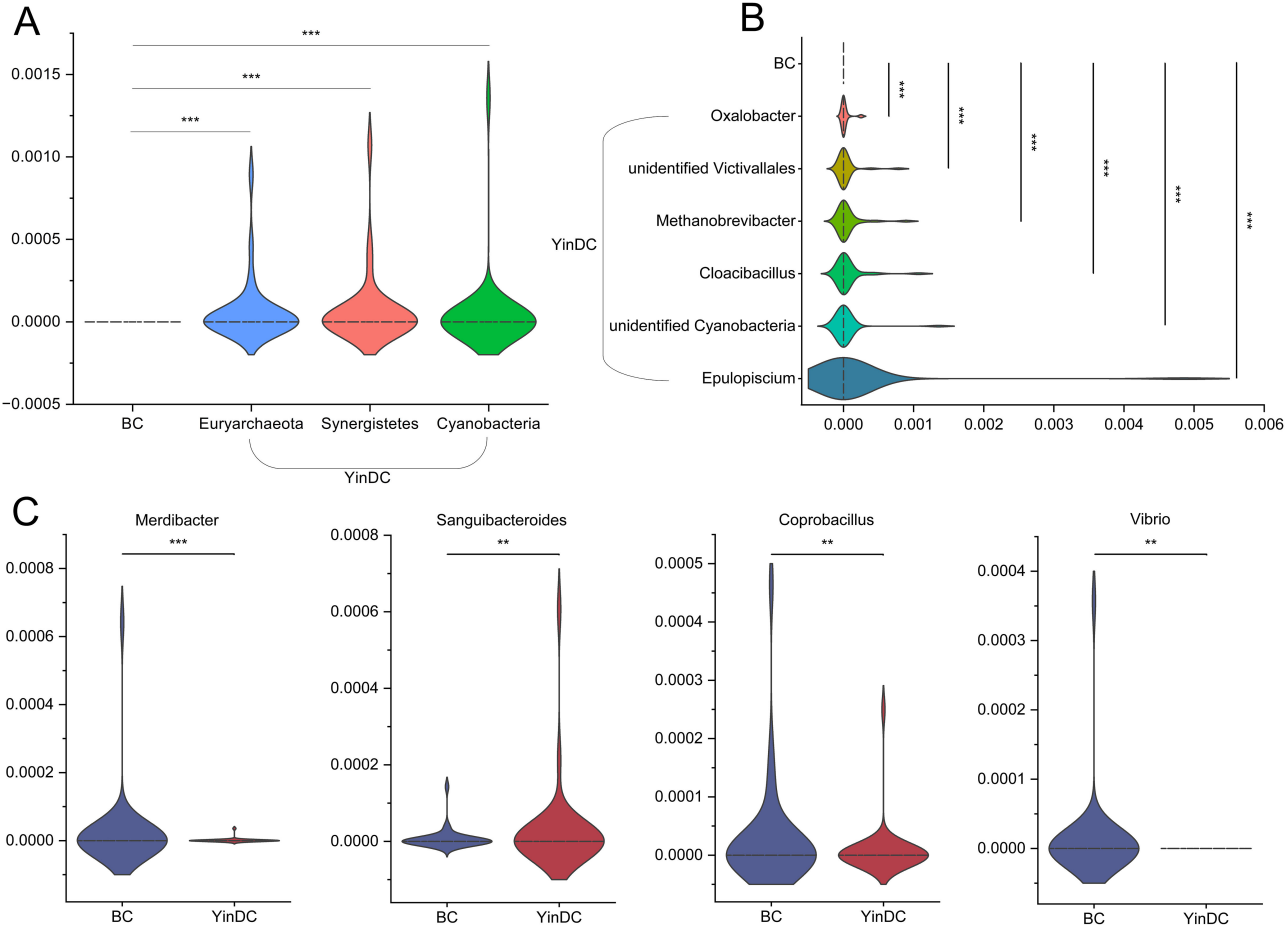
Analysis of the exact differential species of the two groups at the phylum and genus levels with Metastat. All statistical results met q < 0.05. **(A)** Differential species at the phylum level. **(B, C)** Differential species at the genus level. **P<0.01, ***P<0.001.

Metastat analysis identified differential taxa at a significance level of P < 0.001 and q < 0.05: Cyanobacteria, Synergistetes, and Euryarchaeota at the phylum level and *Oxalobacter*, unidentified *Victivallales*, *Methanobrevibacter*, *Cloacibacillus*, unidentified *Cyanobacteria*, and *Epulopiscium* at the genus level were significantly abundant in the YinDC group. Additionally, *Merdibacter*, *Coprobacillus*, and *Vibrio* were less abundant in the YinDC group, while *Sanguibacteroides* was more abundant (**Figure 3**). These results indicated that the richness and diversity of gut microbiota may be associated with the susceptibility of the YinDC women to aging.

### 3.3 Identification of the feature gut microbiota associated with YinDC aging

To comprehensively identify the gut microbiota associated with YinDC aging, we further applied machine learning methods to our study. Firstly, based on the P-values, the top 20 gut microbiota with the largest differences between the YinDC and BC groups were selected according to the P values. Then, we built predictive models based on four machine learning methods, namely, LR, RF, SVM, and XGBoost. Subsequently, we used RF, XGBoost and added Lasso regression analysis to screen the effective gut microbiota influencing YinDC aging. We suggest that the common gut microbiota screened by two or more machine learning methods are closely associated with YinDC aging.

**Figure 4:**
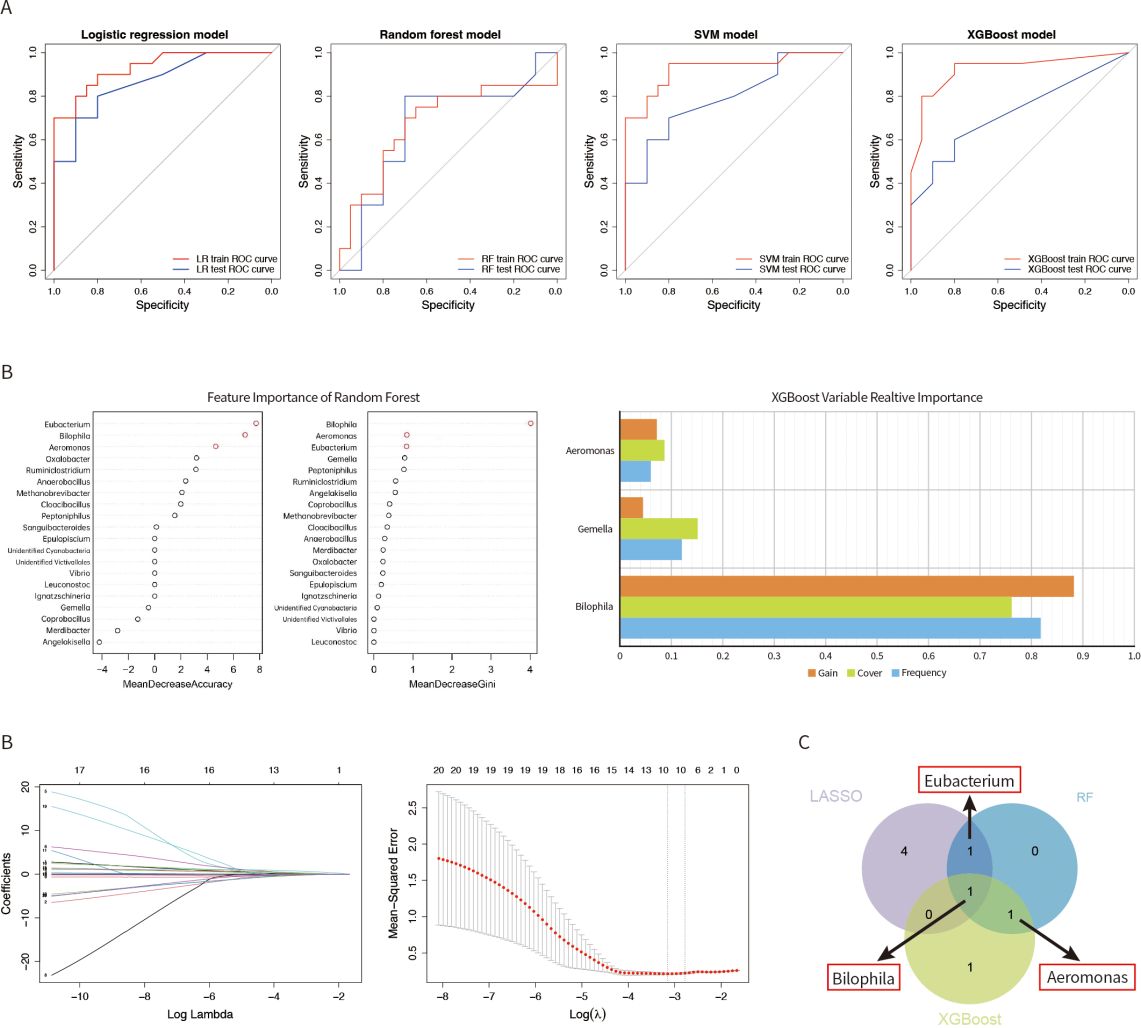
The ROC curves of the predictive models were constructed by four machine learning methods: **(A)** LR, RF, SVM, and XGBoost. **(B)** Importance of variables selection using the RF method. Important variables screened by the XGBoost method. Gut microbial identified by the Lasso. **(C)** Venn diagram showing the gut microbiota intersected by Lasso, RF, and XGBoost.

The results of the model construction were shown in the figure (**Figure 4A**), where the ROC curve was used to evaluate the model prediction capability. From the figures, it can be seen that the prediction performance of the training dataset was higher than that of the test dataset, which is in line with the conventional expectation. The AUC values of the ROC curves in the training dataset were 0.931 for LR, 0.680 for RF, 0.926 for SVM, and 0.924 for XGBoost. In the test dataset, the AUC values were 0.865 for LR, 0.665 for RF, 0.795 for SVM, and 0.725 for XGBoost. Additionally, we presented the validation results of four prediction models in **Table 2**. This table offered a comprehensive overview of the performance metrics for the predictive models developed using these machine learning techniques, including their respective AUC, cut-off values, sensitivity, specificity, and positive predictive values (PPV). The results indicated that the predictive models constructed by the four machine learning methods had good prediction performance, and the accuracy of the predictive model constructed by LR was the best.

**Table 2:** Validation results of four prediction models using LR, RF, SVM, XGBoost.

Model	AUC		Cut-off		Sensitivity		Specificity		PPV	
	Test	Train	Test	Train	Test	Train	Test	Train	Test	Train
LR	0.865	0.931	0.265	0.312	0.800	0.900	0.800	0.800	0.800	0.818
RF	0.665	0.680	0.173	0.305	0.800	0.750	0.700	0.650	0.727	0.682
SVM	0.795	0.926	0.435	0.443	0.700	0.950	0.800	0.800	0.778	0.826
XGBoost	0.725	0.924	0.159	0.427	0.600	0.950	0.800	0.800	0.750	0.826

The results of RF (**Figure 4B**) showed that *Eubacterium*, *Bilophila*, and *Aeromonas* all had highly significant effects on the predicted outcomes for both the effect of features on the model accuracy and the effect of features on the Gini index. The XGBoost method identified the top 3 feature gut microbiota, which were *Bilophila*, *Aeromonas*, and *Gemella*. In lasso regression, the nonzero coefficients were utilized to identify independent predictive features in the training set and can be used to determine the ideal parameter lambda through multi-fold cross-validation. When the nonzero coefficients were 0.011, -0.016, 0.111, -0.011, 0.027, and 0.032, we screened six gut microbiota, namely *Merdibacter*, *Coprobacillus*, *Bilophila*, *Peptoniphilus*, *Eubacterium* and *Ruminiclostridium*. Eventually, we showed the potential indicators of gut microbiota by means of a Venn diagram (**Figure 4C**). We suggest that *Bilophila*, *Eubacterium*, and *Aeromonas* are also closely associated with aging in the YinDC women.

### 3.4 Different functional profiles between the BC and YinDC groups

**Figure 5:**
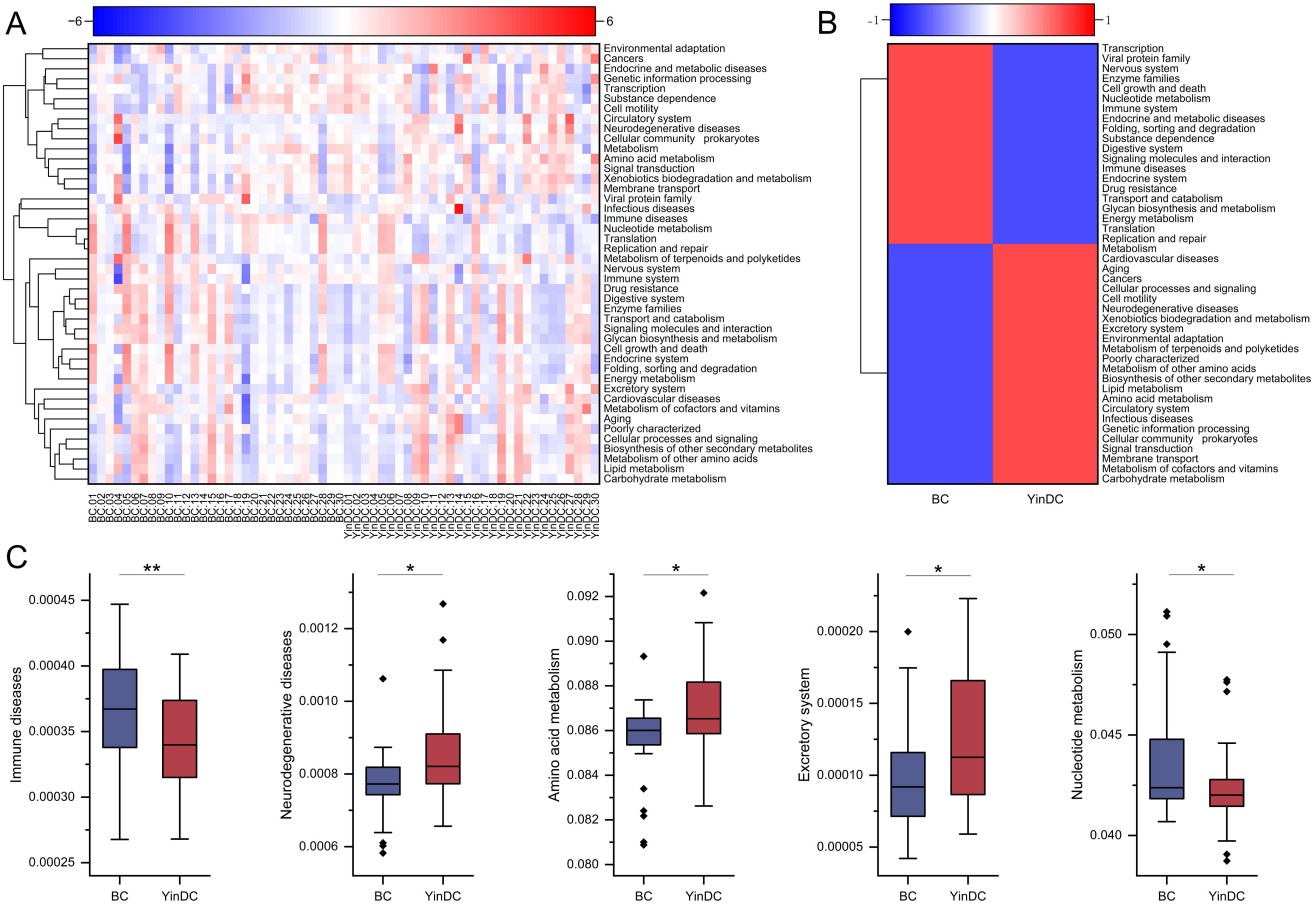
Function prediction with Tax4Fun at level 2. **(A)** Heat map clustered by samples showing 44 predicted functional pathways. **(B)** Heat map clustered by groups showing 44 predicted functional pathways. **(C)** Differential functional pathways between the BC and YinDC groups. The functional pathways are listed in order of P-value. *P< 0.05, **P< 0.01.

Functional prediction analysis was performed with Tax4Fun to characterize the functional profiles of YinDC. At level 2, 44 functional profiles were identified in both the BC and YinDC groups, with 5 showing significant differences (**Figures 5A, B**). Specifically, immune disease (P = 0.006) and nucleotide metabolism (P = 0.049) were down-regulated in the YinDC, whereas neurodegenerative diseases (P = 0.01), amino acid metabolism (P = 0.012), and excretory system (P = 0.017) were up-regulated (**Figure 5C**).

**Figure 6:**
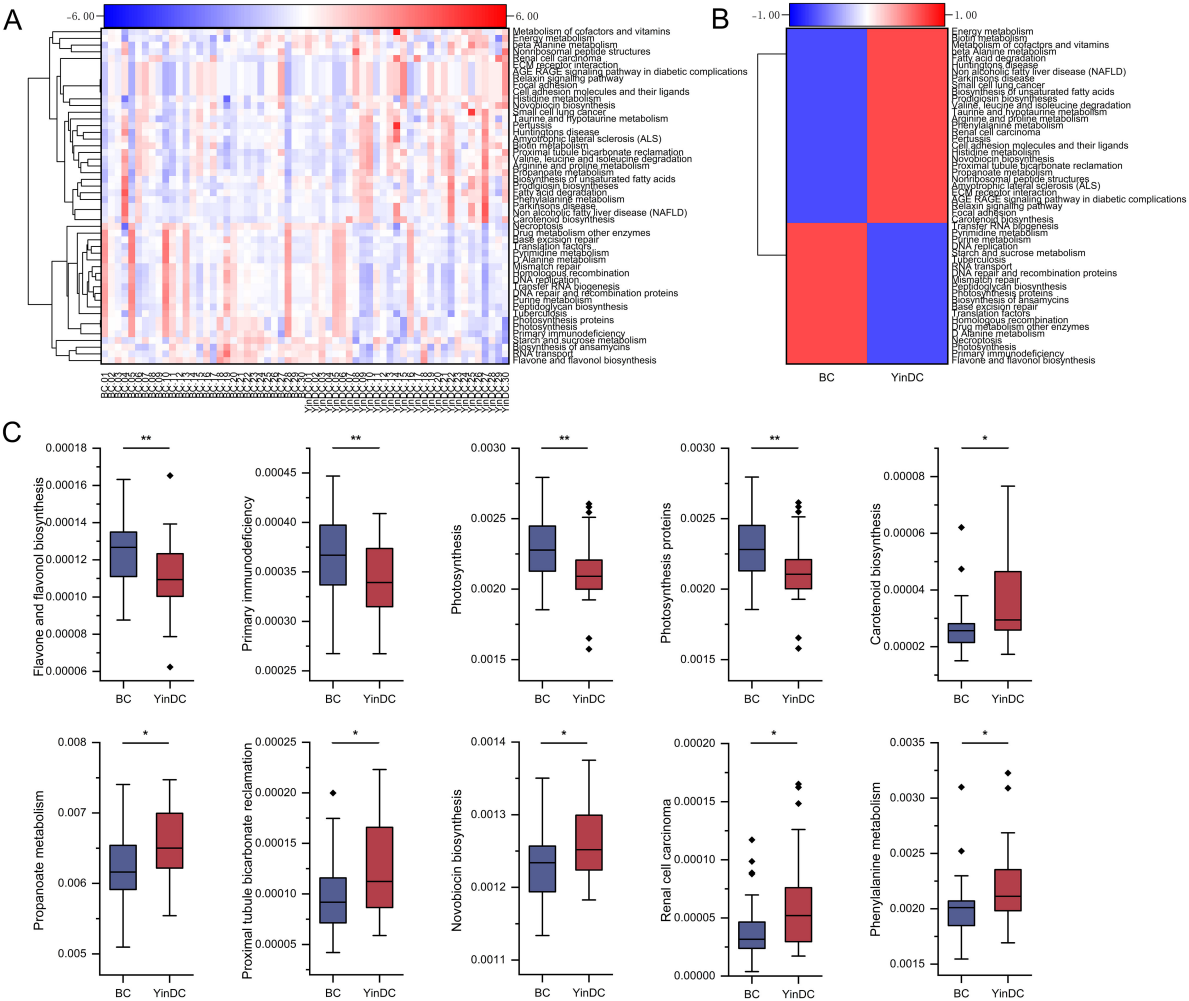
Functional prediction with Tax4Fun at level 3. **(A)** Heat map clustered by samples showing the top 50 predicted functional pathways according to the P-value. **(B)** Heat map clustered by groups showing the top 50 predicted functional pathways according to the P-value. **(C)** Top 10 differential functional pathways between the BC and YinDC groups. The functional pathways are listed in order of P-value. *P< 0.05, **P< 0.01.

In addition, 385 functional profiles were predicted at level 3 for both groups. The top 50 functions were clustered into heat maps based on P-values (**Figures 6A, B**). 25 functions exhibited significant differences between the BC and YinDC groups. The top 10 functions are depicted in **Figure 6C**. Flavone and flavonol biosynthesis (P = 0.004), primary immunodeficiency (P = 0.005), photosynthesis (P = 0.007), and photosynthesis proteins (P = 0.008) were down-regulated in YinDC. Conversely, carotenoid biosynthesis (P = 0.013), propanoate metabolism (P = 0.015), proximal tubule bicarbonate reclamation (P = 0.018), novobiocin biosynthesis (P = 0.019), renal cell carcinoma (P = 0.021), and phenylalanine metabolism (P = 0.021) were up-regulated (**Figure 6C**).

**Figure 7:**
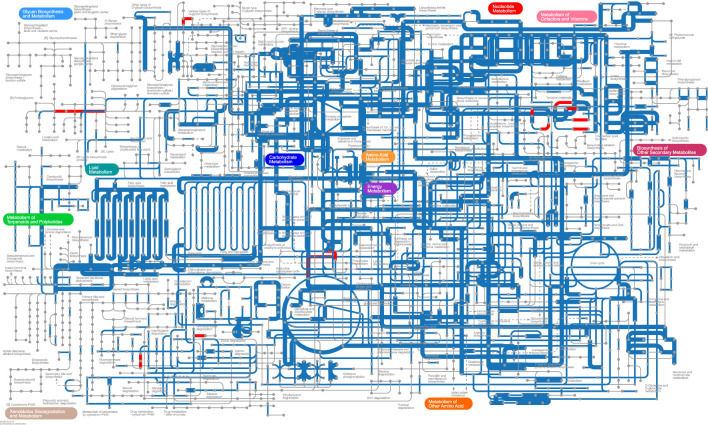
iPath map displaying metabolic pathways of the BC and YinDC groups. The colored lines indicate the presence of pathways. Blue: shared pathways. Red: unique pathways in the YinDC group. The edge width indicates differences in abundance. Wider: higher abundance in the BC group. Narrower: higher abundance in the YinDC group.

We used iPath v3 (Interactive Pathway Explorer v3, http://pathways.embl.de) to visualize the predicted metabolic pathways in the BC and YinDC groups (**Figure 7**). The pathways were mapped based on KOs (KEGG orthologous groups) annotated from the KEGG database. The YinDC group exhibited unique pathways, highlighted with red lines, including lysine degradation (map00310), tyrosine metabolism (map00350), chlorocyclohexane and chlorobenzene degradation (map00361), N-Glycan biosynthesis (map00510), various types of N-Glycan biosynthesis (map00513), arachidonic acid metabolism (map00590), polycyclic aromatic hydrocarbon degradation (map00624), and carbon fixation pathways in prokaryotes (map00720). In contrast, the BC group showed no unique pathways.

### 3.5 Correlations of the gut microbiota and YinDC-characteristic NF-KappaB pathway gene expressions

**Figure 8:**
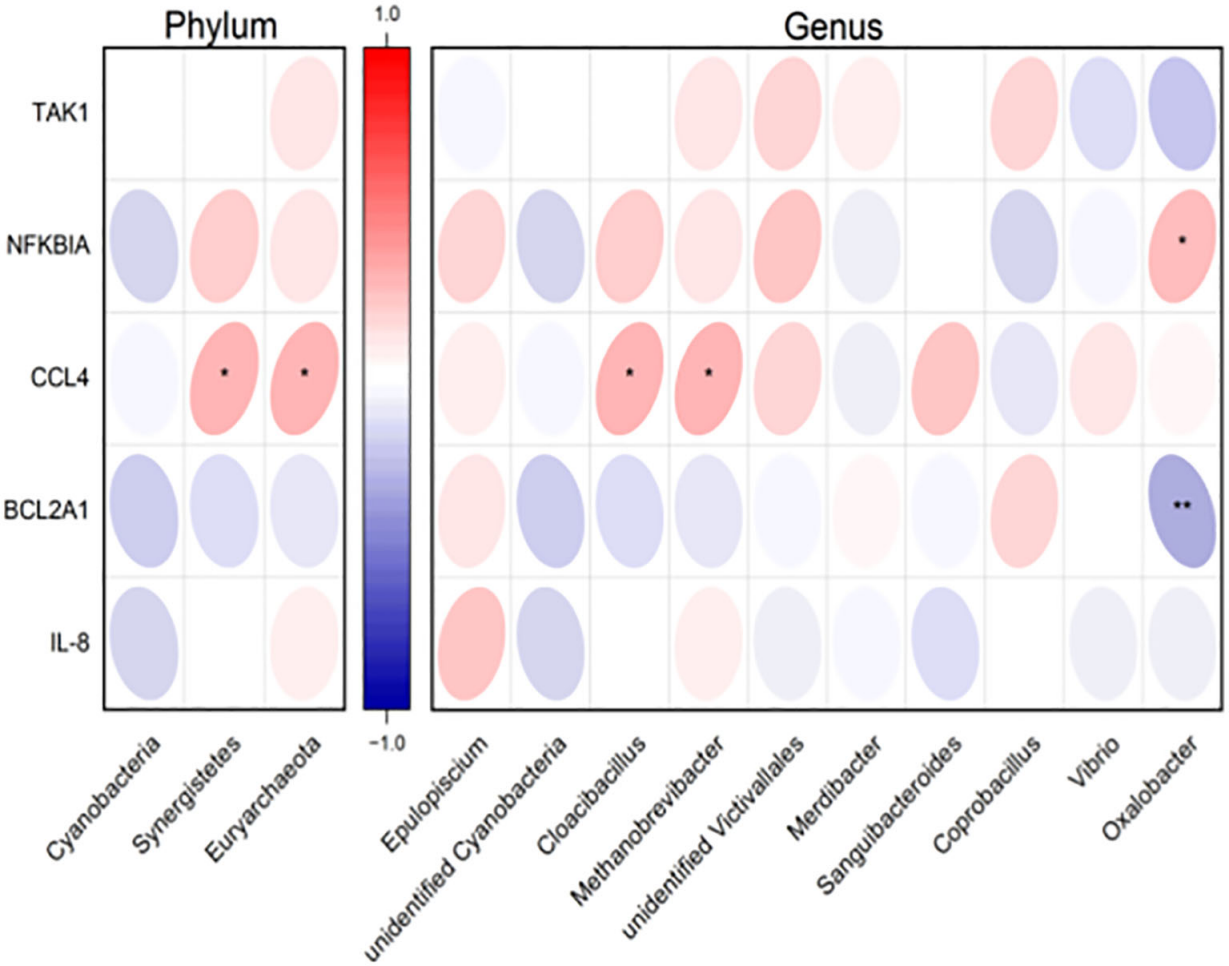
Heatmap displaying the correlation of differential gut microbiota at the phylum and genus levels with YinDC-characteristic NF-KappaB pathway gene expressions. Red: positively correlated. Blue: negatively correlated. *P< 0.05, **P< 0.01.

In our previous study, we aimed to reveal the dysregulation genes in YinDC women, which may underlie the premature aging observed in this constitution, and to explore the molecular mechanisms that make YinDC prone to aging. We discovered that YinDC exhibited dysregulated NF-KappaB pathway gene expressions (Yu et al., 2021). Specifically, TAK1, NFKBIA, CCL4, BCL2A1, and IL-8 mRNAs were abnormally expressed in YinDC and linked to multiple inflammation and immune-related pathways. Among these, NFKBIA and CCL4 mRNAs were up-regulated, whereas BCL2A1 mRNA was down-regulated. To further investigate the relationship between gut microbiota and gene expression, we performed a correlation analysis, with the results visualized in a heat map (**Figure 8**). At the phylum level, Synergistetes (r = 0.313, P = 0.015) and Euryarchaeota (r = 0.311, P = 0.016) showed positive correlations with CCL4 mRNA expression. At the genus level, *Cloacibacillus* (part of Synergistetes, r = 0.313, P = 0.015) and *Methanobrevibacter* (part of Euryarchaeota, r = 0.311, P = 0.016) also correlated positively with CCL4 mRNA. Additionally, *Oxalobacter* genus demonstrated a positive correlation with NFKBIA mRNA expression (r = 0.263, P = 0.042) and a negative correlation with BCL2A1 mRNA (r = -0.339, P = 0.008). These correlations align with prior research, suggesting that variations in gut microbiota composition underlie inflammatory aging in YinDC women.

## 4 Discussion

As research advances in exploring the correlations between TCM constitutions and disease susceptibility, a recent study has uncovered distinct gut microbiota profiles between individuals with phlegm-dampness constitution and non-phlegm-dampness obese individuals, correlating these microbiota variations with metabolic disease risk (Shin et al., 2022). Furthermore, another study has dissected the damp-heat constitution’s influence on the gut microbiota and urinary metabolites in Chinese infants, revealing substantial disparities compared to healthy infants (Zhao et al., 2022). These findings emphasize the significance of TCM constitutions in disease prevention and intervention strategies. Building on these insights, our team, through a meticulous literature review and preliminary studies, has identified an association between the YinDC and the prevalence of age-related diseases. To reveal the biological mechanisms underlying this association, we have conducted this study to investigate the association between the gut microbiota and premature aging in women with YinDC. We aim to fill the gap in the understanding of the susceptibility of YinDC women to premature aging, particularly in the context of gut microbiota. We hope that our research will contribute to the development of new strategies for personalized anti-aging interventions that target the individual’s constitution.

In this study, we employed 16S rDNA gene sequencing of fecal samples to compare the gut microbial communities between the YinDC and BC groups. Our analytical focus was on the composition of the gut microbiota at the phylum and genus levels. We observed significant differences in gut microbiota composition between the two groups. Certain gut microbiota, such as Cyanobacteria, Synergistetes, *Bacteroides*, *Bifidobacterium*, *Haemophilus*, *Alistipes*, and *Dialister*, prevalent in the YinDC group, are closely associated with organismal aging, metabolism, and inflammatory responses. We then further analyzed the functional characteristics of the gut microbiota of the YinDC group and found that compared to the BC group, the YinDC group had the most functional gene pathways related to metabolism. Aging, cancers, and infectious diseases were over-represented, while the nervous system, endocrine system, and immune system were under-represented in the gut microbiota of the YinDC group. This result shows that women with YinDC may suffer from metabolic disorders, decreased neuroendocrine-immune function, and are prone to premature aging, as well as infectious diseases and cancer.

Cyanobacteria has been implicated in premature aging, as evidenced by a study on two distinct mouse models of progeria, which demonstrated intestinal dysbiosis with an increased abundance of Cyanobacteria, suggesting a potential role in the aging process (Bárcena et al., 2019). In our research, the relative abundance of Cyanobacteria was notably higher in the YinDC group compared to the BC group. This provides a microbiome-based indicator for the susceptibility of YinDC women to aging.

Studies show that infectious diseases are often associated with inflammatory responses (Camps et al., 2017), and the elderly are among the susceptible and high-risk groups for infections. Almost every living organism experiences dysregulation of inflammation with age. This state of chronic inflammation that correlates with aging is sometimes referred to as inflame-aging. It is a high-risk factor for the occurrence, progression, and complication of many chronic diseases, including cardiovascular diseases and neurodegenerative diseases (Bektas et al., 2018) Inflammatory aging determines the aging process’s rate and the organism’s lifespan (De Martinis et al., 2005). The results of our study showed a positive correlation between Synergistetes and CCL4 mRNA (0.01≤P<0.05) as well as between Dialister etc. and NFKBIA mRNA (P<0.01). In the early stage, our genomics study found an enhanced inflammatory response in the YinDC group, which was closely related to the NF-κB inflammatory signaling pathway and contributed to by the expression of NFKBIA and CCL4 mRNA (Yu et al., 2021). Thus, we conclude that the existence of Synergistetes and *Dialister* etc. may activate the NF-κB pathway and lead to inflammatory aging.

At the same time, we found that microbiota with relatively high abundance in the YinDC group as *Bacteroides*, Synergistetes, *Haemophilus, Alistipes*, and *Dialister*, were all involved in the body’s inflammatory response in this study. Among Bacteroides species, Bacteroides fragilis strains are opportunistic pathogens. *Enterotoxigenic Bacteroides Fragilis* (*ETBF*), a kind of Bacteroides fragilis strain, elicit intestinal secretion and induces intestinal inflammation in animals and humans (Sears, 2009). Members of the phylum Synergistetes have frequently been detected in the human oral cavity at sites of dental disease (Vartoukian et al., 2009). The study by Zhang S et al. found that periodontitis communities had higher proportions of Synergistetes (Zhang et al., 2020). *Haemophilus* is closely related to a variety of inflammatory and infectious diseases. The encapsulated species of *Haemophilus* influenzae (especially type b, Hib) caused a variety of invasive diseases such as meningitis, epiglottitis, and bacteremic pneumonia. In contrast, nonencapsulated (non-typeable) H. influenzae (NTHi) species were traditionally linked to noninvasive infections, such as otitis, sinusitis, conjunctivitis, and nonbacteremic pneumonia (Cohen et al., 2019). Metagenomic studies of fecal samples from the mouse model of spontaneous CD ileitis revealed the enrichment of *Alistipes* compared to the parental AKR/J mouse colony, suggesting that *Alistipes* spp. may be associated with the promotion of segmental ileitis (Parker et al., 2020; Rodriguez-Palacios et al., 2015). Some researchers have found that *Dialister* was significantly correlated with systemic inflammatory cytokines (Ling et al., 2016; Tito et al., 2017), such as Ankylosing Spondylitis Disease Activity Score. Besides, carrying proteobacteria may drive and trigger instigate chronic colitis (Carvalho et al., 2012). We can speculate that this alteration in the gut microbiota may cause YinDC women to be more susceptible to progeria through inflammatory aging and metabolic aging. In addition, this study found that metabolic pathways unique to the negative deficiency group include arachidonic acid metabolism (map00590: Arachidonic acid metabolism). The arachidonic acid metabolic pathway plays an important role in the synthesis of inflammatory mediators, which are mediated by various inflammatory factors and have an important effect on the whole process of inflammation (Ren et al., 2020).

Metabolic diseases play a pivotal role in aging and degeneration. In this study, we found that compared to the gut microbiota of the BC group, Lipid metabolism was over-represented in the gut microbiota of the YinDC group, as Energy metabolism, Amino acid metabolism, and Nucleotide metabolism were under-represented. Amino acid metabolism disorders can induce premature ovarian failure (Qi et al., 2021), which was found relevant to the presentation of YinDC in our preliminary study. In this study, the relevant abundance of bacterial groups involved in human metabolisms, such as Synergistete, increased in the YinDC group. Relevant studies have shown that *Bifidobacteria*, Synergistetes, and their offspring showed a significant increase in relative abundance in diabetes (Biassoni et al., 2020), suggesting some of the characteristic gut microbiota in the YinDC group are closely related to the occurrence of metabolic diseases. Metabolic disorders also contribute to aging (Deshpande et al., 2020; Goldsworthy and Potter, 2014; Hou et al., 2018; Tang and Chua, 2010). Studies show that metabolic functions, including glucose metabolism, lipid metabolism, and energy metabolism, decline with age (Liu Z. et al., 2020). Previous studies have also confirmed that people with YinDC suffer from low levels of energy metabolism and increased glucose metabolism (Li, 2009). Thus, we suspect YinDC people with metabolic disorders are prone to aging.

Cardiovascular disease (CVD) and cancer are also closely related to aging. Studies have shown that colorectal cancer is closely associated with gut *dysbacteriosis*, and *Alistipes* was identified as a potential pathogen that may lead to colorectal cancer (Hasan et al., 2022; Parker et al., 2020). Meanwhile, *Alistipes* is also a risk factor for CVDs such as hypertension, as well as several CVDs such as atrial fibrillation (AF), congestive heart failure (CHF), and atherosclerosis cardiovascular disease (ACVD) (Jie et al., 2017). In our study, the relative abundance of *Alistipes* increased in the YinDC group, suggesting increased risks of cardiovascular disease and cancer among YinDC people. Aging is closely related to neurological, endocrine, and immune system functions. Besides, neurological, endocrine, and immune system disorders exist in the YinDC group. The imbalances in neuroendocrine-immune regulatory networks accelerate the aging process (Yao and Li, 2007).

Furthermore, to explore the potential gut microbiota as fecal indicators associated with YinDC aging, this study constructed the LR, RF, SVM, and XGBoost models. Our results showed that the AUC values of the four models for predicting the YinDC aging were high, with the top value reaching 0.931, indicating that all models had pretty predictive ability. Notably, the LR model had the highest prediction accuracy and also offered good model interpretability. *Bilophila*, *Eubacterium*, and *Aeromonas* were identified as effective gut microbiota associated with YinDC aging by three machine learning methods. These results suggest that the construction of machine learning models can better reveal the association between the gut microbiota and YinDC aging, and *Bilophila*, *Eubacterium*, and *Aeromonas* can be used as potential fecal indicators for predicting premature aging in YinDC individuals.

All these results suggest that the particular changes in the gut microbiota of YinDC women may be the reason they are susceptible to pre-mature aging. Regarding the microbiome-based results, YinDC, based on TCM constitution classification models, is certified as an appropriate carrier for aging-related studies. This recognition may pave the way for novel perspectives in anti-aging research and underscores the unique contribution of TCM to the field of aging studies.

This study, while informative, is not without its limitations. Firstly, compared to 16S rDNA gene sequencing, shotgun metagenomic sequencing has a higher sequencing depth, which can identify more species of bacteria and provide comprehensive microbial gene content data. Secondly, our current investigation was a single-center, cross-sectional study with a relatively small sample size, which may constrain the generalizability of our results. Future multi-center studies with larger cohorts are warranted to validate and extend the findings presented here. Thirdly, this research was confined to assessing the abundance of gut bacteria associated with YinDC aging; thus, future in-depth studies are essential to elucidate the mechanisms through which gut microbiota modulate the aging process. Lastly, while this study concentrated on the association between YinDC and aging, it is acknowledged that factors such as BMI and dietary habits also play a significant role in aging by influencing gut microbiota composition. Consequently, future research should incorporate a more holistic set of influencing factors to provide a comprehensive understanding of the aging process.

## 5 Conclusions

In this study, we conducted a cohort study including 60 women with YinDC and BC to analyze their gut microbiota composition. We integrated traditional gut microbiota analysis with machine learning methods to reveal the association between gut microbiota and premature aging in YinDC women. Our findings revealed that the composition and functional characteristics of the gut microbiota were significantly associated with premature aging in these women. Identifying effective aging-related gut microbiota indicators can be beneficial for guiding personalized anti-aging interventions.

## Data Availability

The raw data supporting the conclusions of this article will be made available by the authors, without undue reservation.
